# Reliability and validity of the German translation of the de Morton Mobility Index (DEMMI) performed by physiotherapists in patients admitted to a sub-acute inpatient geriatric rehabilitation hospital

**DOI:** 10.1186/s12877-015-0035-y

**Published:** 2015-05-03

**Authors:** Tobias Braun, Ralf-Joachim Schulz, Julia Reinke, Nico L van Meeteren, Natalie A de Morton, Megan Davidson, Christian Thiel, Christian Grüneberg

**Affiliations:** Department of Applied Health Sciences, Physiotherapy Program, Hochschule für Gesundheit, Universitätsstr. 105, 44789 Bochum, Germany; University of Cologne, Medical Faculty, Kerpener Str. 62, 50937 Cologne, Germany; Department of Geriatric Medicine, St. Marien-Hospital, Kunibertskloster 11-13, 50668 Cologne, Germany; Catholic Clinic Bochum, Ruhr-University Bochum, Marien-Hospital Wattenscheid, Parkstr. 15, 44866 Bochum, Germany; Health~Holland, Topsector Life Sciences and Health, 2509 The Hague, The Netherlands; CAPHRI, Maastricht University, Minderbroedersberg 4-6, 6211 LK Maastricht, The Netherlands; Donvale Rehabilitation Hospital, Ramsay Health, 1119 Doncaster Road, Donvale, VIC 3111 Australia; School of Allied Health, College of Science, Health and Engineering, La Trobe University, Melbourne, VIC 3086 Australia

**Keywords:** Aged, Mobility limitation, Geriatric assessment, Psychometrics, Rehabilitation

## Abstract

**Background:**

Mobility is a key outcome in geriatric rehabilitation. The de Morton Mobility Index (DEMMI) is an internationally well-established, unidimensional measure of mobility with good psychometric properties. The aim of this study was to examine the reliability and construct validity of the German translation of the DEMMI in geriatric inpatients.

**Methods:**

This cross-sectional study included patients admitted to a sub-acute inpatient geriatric rehabilitation hospital (reliability sample: N = 33; validity sample: N = 107). Reliability, validity, and unidimensionality were investigated.

**Results:**

Inter-rater reliability between two graduate physiotherapists was excellent, with intra-class correlation coefficient of 0.94 (95% confidence interval: 0.88-0.97). The minimal detectable change with 90% confidence was 9 points. Construct validity for the DEMMI was evidenced by significant moderate to strong correlations with other measures of mobility and related constructs (Performance Oriented Mobility Assessment: rho = 0.89; Functional Ambulation Categories: rho = 0.70; six-minute walk test: rho = 0.73; gait speed: rho = 0.67; Falls Efficacy Scale International: rho = −0.68). Known-groups validity was indicated by significant DEMMI mean group differences between independent versus dependent walkers and walking aid users versus non-users. Unidimensionality of the German DEMMI translation was confirmed by Rasch analysis.

**Conclusions:**

The German translation of the DEMMI is a unidimensional instrument producing valid and reproducible measurement of mobility in an inpatient geriatric rehabilitation setting.

## Background

Mobility limitations are common in older people undergoing geriatric rehabilitation [1]. Poor mobility has a crucial impact on older people’s activities of daily living (ADL), participation in social life, fall risk and quality of life [2-4]. Thus, improvement or maintenance of sufficient mobility are important inter-professional goals in geriatric rehabilitation. Health professions are recommended to use outcome measures that are sufficiently reliable and valid for monitoring a patient’s mobility [4].

The World Health Organisation’s International Classification of Functioning (ICF) classifies ‘mobility’ as one of nine domains of ‘activity and participation’ and gives the definition of “moving by changing body position or location or by transferring from one place to another, by carrying, moving or manipulating objects, by walking, running or climbing, and by using various forms of transportation” [5]. In geriatric care, mobility is recognized as an important indicator of the health status of older patients [6].

Like many other western countries [7], Germany is affected by demographic changes, leading to increasing numbers of frail older people needing inpatient rehabilitation during and/or after age-related diseases and/or major life events [8]. Common assessments of mobility, mostly as part of the comprehensive geriatric assessment [9], are the Performance Oriented Mobility Assessment (POMA) [10], the Functional Ambulation Categories (FAC) [11], gait speed measures and the six-minute walk test (6MWT) [12]. The POMA and the FAC use ordinal based scores which have crucial disadvantages compared to interval and ratio level based outcome measures [13,14]. Furthermore, limitations have been reported on the POMA’s reproducibility [15,16]. Gait speed and the 6MWT are ratio level measures, but only assess one single aspect of mobility [17]. A significant number of geriatric inpatients is known to be non-ambulatory initially and they cannot perform these tests at all [18]. Single component mobility measures are therefore invalid to monitor mobility over the whole mobility spectrum in some patients due to floor- and ceiling-effects [17].

Two systematic reviews have outlined the psychometric limitations of existing mobility measures in inpatient and community-dwelling older adults [17,19]. These findings led to the development, using Rasch analysis, of the de Morton Mobility Index (DEMMI), an interval level outcome measure of older people’s mobility [20-23]. Relevant aspects of mobility are assessed with 15 different DEMMI items, evaluating elementary aspects of bed and chair mobility, ambulation as well as static and dynamic balance. The psychometric properties have been examined extensively in acute [20,24] and sub-acute [25] settings as well as in various health conditions [26,27]. The DEMMI is a bedside assessments that can be administered within 10 minutes without the use of special materials and the Australian English and Dutch versions have proven to be sufficiently reliable, valid and responsive measures of mobility in the Australian and Dutch context, respectively [20,24,27].

The Australian English original version has recently been translated into German [28]. A gold standard forward-backward translation method was used to develop a preliminary German DEMMI version, which was then administered by several physiotherapists in a geriatric hospital for three weeks on all incoming patients (n = 133). The process of translation and cross-cultural adaption followed the recommendations given by Beaton et al. [29] and was published in detail elsewhere [28]. Good feasibility was reported by the physiotherapists and only minor changes were made to the instruction form, all in consultation with the test developer (N.A.d.M.). Based on this process, a final German DEMMI version has been scientifically produced, but not yet examined for unidimensionality, validity and reliability. Thus, the aim of this study was to examine these psychometric properties of the German DEMMI version in a geriatric rehabilitation sample in order to further complete the process of cross-cultural validation.

## Methods

This cross-sectional study examined the DEMMI’s psychometric properties in a convenience sample of sub-acute geriatric inpatients treated in a geriatric rehabilitation hospital in Bochum, Germany. Recruitment was initialized by the hospital physiotherapists, who were aware of the inclusion and exclusion criteria and who reported potentially eligible patients to the research coordinators. These patients were then screened for eligibility and invited to participate. The study was approved by the Ethical Review board of the German Confederation of Physiotherapy (registration number: 2012–05). All included participants provided written informed consent.

### Reliability sample

Relative and absolute inter-rater reliability were examined between two physiotherapists with 5 and 7 years of work experience (T.B. and J.R.), respectively. Both assessors were familiar with the DEMMI as they discussed the test instructions and did some pilot-measures in five geriatric patients each prior to the reliability study. Both assessors independently performed the DEMMI in a sample of geriatric inpatients. Both DEMMI measures were performed within 30 minutes and a 10-minute rest was given between the assessments. This was done to create a stable test-retest situation. In a random order, each assessor was the first assessor in half the patients. Both physiotherapists were blind to the results of the other. The test conditions were similar for both measurements with respect to the environment (patient’s room).

Participants were a sample of convenience, that is, inpatients in a German geriatric hospital who were eligible on three randomly selected recruiting days during a period of 3 weeks. Participants were excluded if they had severe dysphasia, documented contraindications to mobilizations or severe cognitive impairment. Patients isolated for infection and to whom death was imminent were also excluded. The presence of any of these exclusion criteria was pre-defined by clinical judgement of the treating physiotherapists and if needed in consultation with the ward physician.

The sample size approximation was based on an inter-rater reliability estimate for the DEMMI of r = 0.87 between two physiotherapist in the sub-acute hospital setting found by others [25]. Following the method presented by Bonett [30], given 2 raters, a planning value of ICC = 0.87 and a desired 95% confidence interval (CI) with the width of 0.20, a minimum sample size of 29 participants was needed.

### Validity sample

The DEMMI’s validity was examined in a sample of geriatric rehabilitation inpatients. Exclusion criteria were the same as for the reproducibility sample, with Mini Mental State Examination (MMSE) scores <21 points and age <60 years. Written informed consent, socio-demographic variables, MMSE, the age adjusted Charlson Comorbidity Index (CCI) and Falls Efficacy Scale International (FES-I) were collected in a first session by a physiotherapist or undergraduate research assistants. In a second session, the DEMMI and other performance based measures of mobility and ambulation (POMA, FAC, 6MWT, gait speed) were performed by one of three different well experienced physiotherapists (T.B., J.R. and a third assessor with 8 years of work-experience) in a standardized order, starting with the DEMMI in each session. All assessors were trained in the administration of the outcome measures.

### Unidimensionality sample

The independent reliability and validity data samples were pooled in order to enlarge the data sample size for subsequent Rasch analysis.

### Measures of mobility

The DEMMI consists of 15 items [20]. The patient is asked to perform mobility tasks in several positions (bed, chair, stand, walk), which the examiner rates on 2- or 3-point response options, resulting in a maximum ordinal score of 19 points. A conversion table allows for transformation of the raw score into a total interval DEMMI score, which ranges from 0 to 100 points, with higher scores indicating a higher level of mobility. The DEMMI has a hierarchical structure, and thus each assessed individual can be located on the 101 point mobility spectrum. The DEMMI form consists of one paper sheet, with the items printed on one side and the instruction protocol on the other, which makes it easy to use in clinical practice [20,28]. The German DEMMI and a German instruction handbook can both be downloaded free of charge (www.hs-gesundheit.de).

The POMA is a clinician-observed measure of mobility and fall risk, consisting of 2 sub-scales (balance and gait) [10,31]. A maximal total score of 28 points can be reached, with higher scores indicating higher mobility functions. Although results of reproducibility are inconclusive [15,16,32], it is considered to be a valid measure of older people’s fall risk and mobility [10,31,32].

The clinician-completed FAC rates the level of independence and functional ambulation over a walking distance of 10 meters on a 6-point ordinal scale [11,33,34]. Lower scores, where physical assistance is needed, indicate poorer mobility than higher scores, where the patient is able to ambulate independently.

For the 6MWT [12], the test subject is asked to walk along a plain walkway for 6 minutes. The distance in meters is measured, with longer distances indicating a better walking capacity and higher velocity. Walking aids were allowed and breaks were offered if needed. The 6MWT is a reliable and valid instrument to quantify mobility and walking endurance in older individuals [35,36]. In non-ambulatory participants, the 6MWT was scored as 0 meters.

Comfortable gait speed was assessed over a distance of 10 meters [37]. The time measurement started after a gait initiation phase of some steps [38] and participants were allowed to use their usual walking aid. Distance and time were measured with a measuring wheel and a stop-watch, respectively. In order to reduce burden on the participants, measurements were taken during the 6MWT performance. Gait speed can be measured reliably and it is a valid measure of mobility and health status of older people [37,39]. Participants who could not ambulate without physical assistance, or those who needed >90 seconds, were scored as non-ambulatory.

The FES-I is one of the most commonly-used measures of fear of falling [40,41]. The person is asked to rate his or her concerns regarding falling while performing several ADL situations on a 4-point Likert-scale (“not at all concerned” to “very concerned”). Most questions deal with concerns in mobility activities (such as getting in or out of a chair, walking around in the neighbourhood, walking on an uneven surface). Scores range from 16 to 64 points, with higher values representing more concerns in fall-prone situations. As there is a strong correlation between fall risk, ambulation and mobility [42,43], a German version of the FES-I was administered by interview as a reproducible and valid self-reported instrument for construct validity analysis [40,44,45].

### Statistical analysis

Data were analysed using SPSS 21.0 for all analyses except for the Rasch analysis, which was completed using RUMM2030. Descriptive statistics were used to present sample characteristics. Interval-based data were examined for normal distribution with the Shapiro-Wilk test of normality and by visual inspection of the related histograms and p-p-plots. A *P*-value <5% indicated statistical significance in all performed analysis.

#### Reliability

Inter-rater reliability was examined using the intra-class correlation coefficient (ICC) model 2.1 (two-way random effects model) [46,47]. Type of disease, as potential confounding factor, was analysed by a visual scatter plot inspection. A uniform distribution of points without formation of disease groups (ICD-10 categories: musculoskeletal, circulatory, respiratory, nervous system or digestive, based on the primary diagnosis given by the ward physician) would indicate no confounding by the factor “type of disease”.

The minimal detectable change (MDC) with 90% confidence, a quantification of absolute agreement, was calculated as √2 x standard error of measurement (SEM), multiplied by 1.64. The SEM was calculated as the pooled standard deviation (SD) x √(1-ICC). MDC_90_ is defined as the minimal amount of change that needs to occur between repeated assessments in an individual to exceed, with 90% confidence, the error of the measurement [48]. The method of Bland and Altman was used to illustrate agreement between the two raters [49]. Differences between raters were plotted against their mean score. Thus, points scatter around a horizontal mean difference line, which should be close to zero within the upper and lower 95% limits of agreement (ie, mean difference ±1.96 SD of the difference). Cronbach’s alpha, a measure of internal consistency, was derived from the validity sample due to the larger sample size [50].

#### Validity

Convergent, discriminant and known-groups validity were examined as different aspects of the DEMMI’s construct validity. Correlations between the DEMMI and other functional measures were calculated with Spearman’s correlation coefficient rho (ordinal) and Pearson’s correlation coefficient r (interval) together with the appropriate 95% CIs [51]. We hypothesized that the DEMMI would show a very strong (≥.80) correlation with a multi-component mobility scale (POMA) and a strong (≥.70) correlation with outcome measures of ambulation alone (FAC, gait speed, 6MWT). The FES-I is a patient reported measure of fear of falling during performance of ADLs, a construct considered to be related to mobility-perceptions, but not as strongly as outcome measures of performance of ambulation. Therefore, we hypothesized a negative moderate correlation (−0.50 to −0.69) between DEMMI and FES-I scores. The hypothesis with respect to discriminant validity was a non-significant, low correlation between DEMMI scores and measures of comorbidity and cognition (CCI and MMSE, respectively).

For known-groups validity, we hypothesized that participants ambulating without a walking aid would have significantly higher DEMMI scores than participants using a walking aid (Mann–Whitney *U* test, P < 0.05). The difference between the mean scores of both groups was assumed to exceed the minimal clinical important difference (MCID) of 10 DEMMI points reported for the Australian English DEMMI version [20]. Furthermore, three groups with respect to the self-reported level of dependence in in-hospital ambulation were defined (non-ambulatory, ambulatory with assistance and independent ambulation). It was hypothesized that mean DEMMI scores would be higher in participants mobile with assistance than in non-ambulatory ones, and that independent ones had the highest scores. Mean group differences, which were hypothesized to be larger than 10 DEMMI points, were investigated by the use of a Kruskal-Wallis test with *post hoc* analysis between groups (Mann–Whitney *U* test with corrected *P* < 0.017) [52].

#### Rasch analysis

The English DEMMI version was developed based on the Rasch model [23] in 106 Australian older acute medical patients (81.2 ± 7.3 years of age, 47% female) [20]. Data fitted the model in various conditions such as patients with hip fracture, older acute medical patients and older patients with knee or hip osteoarthritis [20,27,53]. The Rasch model is a probabilistic model that asserts that item response is a logistic function of item difficulty and person ability [23]. Rasch analysis was conducted in this study to complete the cross-cultural validation process for the German version of the DEMMI.

Overall fit to the model was evident if item trait interaction chi-square *P* was greater than 0.05 and item fit was indicated by fit residuals less than ±2.5 and a non-significant Bonferroni adjusted Chi-square *P* value. Local independence of items is an assumption of the Rasch model. Local dependence occurs when the response to one item is dependent on the response to another and can inflate the apparent internal consistency of the scale. The assumption of local independence of items was checked by identifying any items with person-item residual correlations larger than 0.2. A subtest analysis using the correlated items was then undertaken to determine whether the internal consistency (Personal separation index and Cronbach alpha) of the whole item set was higher than for the subtest. The assumption of unidimensionality (all items reflecting a single underlying latent trait) was tested by creating subsets of items with the most different loadings on the residual principal components analysis. Paired t-tests were conducted on the estimates of person abilities generated using the item subsets and fewer than 5% of cases with significantly different scores (P < 0.05) indicates a unidimensional scale [54].

Differential Item Functioning (DIF) is a form of item bias that occurs when persons of the same ability perform differently on an item based on another variable. In this study, DIF for the DEMMI was investigated for age (<80 years and 80+ years), gender and age-adjusted CCI score (0–6 and 7+). A target sample size of at least 100 up to 144 was set for this study to provide 95% confidence within ±0.5 logits [55].

## Results

**Figure 1:**
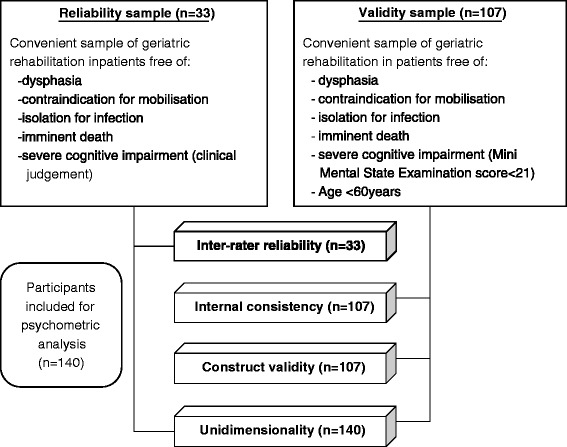
Flow chart of study samples and psychometric analyses.

A flow chart of the included samples and their inclusion in psychometric analysis is given in Figure 1.

### Inter-rater reliability

Thirty-three participants were assessed twice by the two physiotherapists. Most of the participants were female (n = 22, 61%), the mean age was 79.5 ± 7.3 years and causes for rehabilitation were mainly musculoskeletal (53%), circulatory (19%) or respiratory (6%) diseases. DEMMI scores of both raters were normally distributed (W = 0.956; p = 0.200 and W = 0.971 p = 0.508). The mean scores were 51 ± 16 (8–85) and 50 ± 15 (8–74), with a mean difference of 1.7 (95% CI: −0.2 to 3.5). The ICC_2,1_ between both raters was 0.94 (95% CI: 0.88 to 0.97). There was no confounding by the factor “type of disease”.

**Figure 2:**
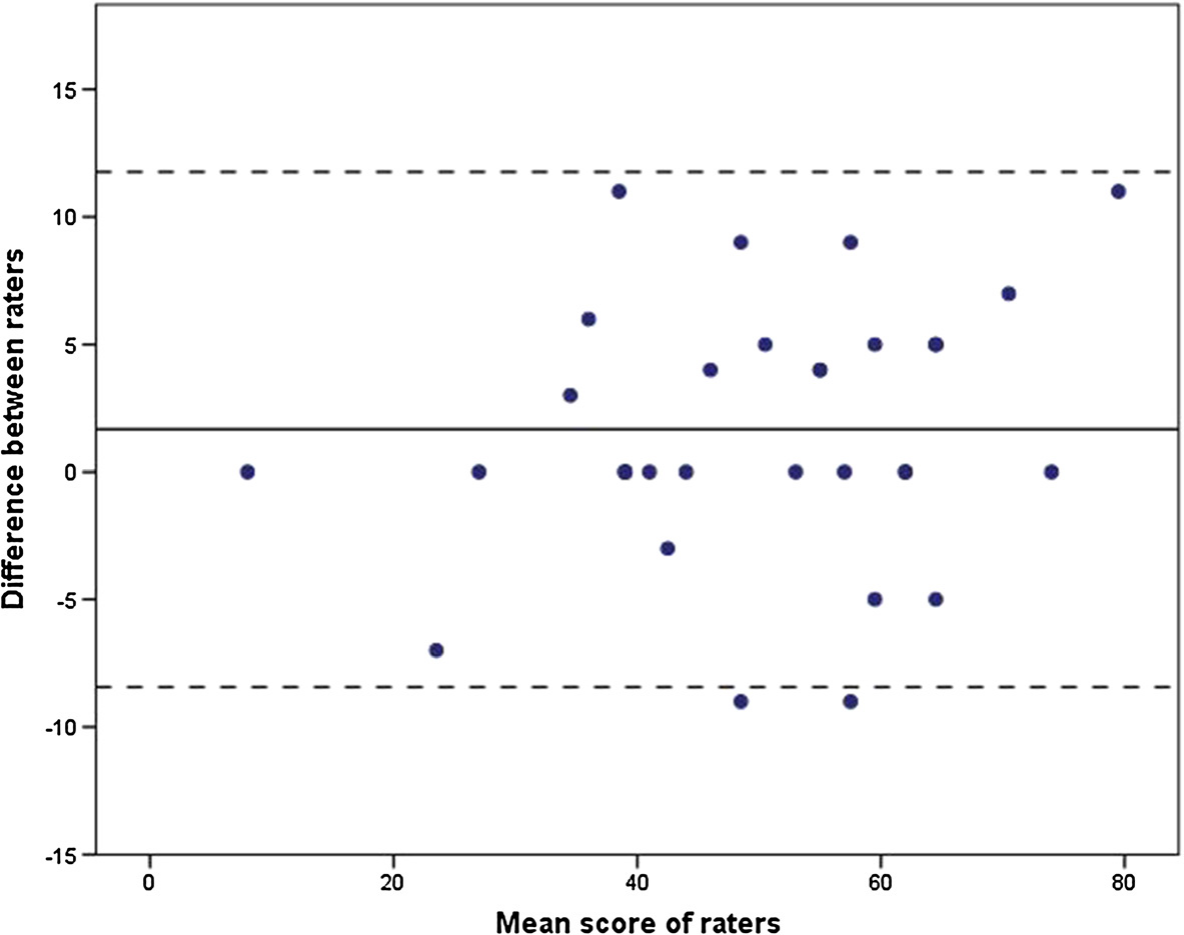
Bland-Altman plot. The x-axis represents the mean sores of the raters and the y-axis represents the difference between both raters. The straight line represents the mean difference between both measures; dotted lines represent the 95% upper and lower limits of agreement.

Absolute agreement (MDC_90_) was 8.8 points on the 100-point DEMMI scale, based on a pooled SD of 15.6 and a SEM of 3.8. Table 1 shows the absolute percentage of agreement per item, which varied between 73% and 100%. The Bland-Altman plot is illustrated in Figure 2. The upper and lower 95% limits of agreement were 11.8 and −8.4, respectively.

**Table 1:** **Absolute percentage of agreement between the 2 raters per DEMMI item**

**No.**	**Item**	**Agreement (%)**
1	Bridge	91
2	Roll onto side	85
3	Lying to sitting	73
4	Sit unsupported in chair	100
5	Sit to stand from chair	91
6	Sit to stand without using arms	94
7	Stand unsupported	97
8	Stand feet together	97
9	Stand on toes	100
10	Tandem stand with eyes closed	94
11	Walking distance	85
12	Walking independence	85
13	Pick up pen from floor	85
14	Walk 4 steps backwards	94
15	Jump	91

### Construct validity

Participant’s characteristics and outcome values are presented in Table 2. A total of 107 patients participated, 65% of them were female. Seventy-nine percent of participants walked independently in the hospital, most of them using a walking aid, but some (13%) reported to be non-ambulatory at all. Ninety-nine participants were able to perform the gait speed measure over 10 meters.

**Table 2:** **Validation sample characteristics**

**Characteristic (n = 107)**	**Value**
Age (years)	80 ± 6 (64–97)
Male/female (%)	35/65
Charlson Comorbidity Index (age adjusted)	6 (5–7)
Mini Mental State Examination	29 (27–30)
Diagnosis (ICD-10 categories, %)	
Musculo-skeletal	58
Circulatory	11
Nervous system	8
Digestive	6
Other	17
Time between admission and anamnesis (days)	13 ± 6 (1–27)
Time between anamnesis and assessment (days)	1.5 ± 1 (0–7)
Time between assessment and discharge (days)	9 ± 6 (0–25)
Duration of inpatient stay (days)	24 ± 6 (9–42)
In-hospital mobility - state (self-reported)	
Independent/with supporting person/non-ambulatory, n (%)	85/8/14 (79/8/13)
In-hospital mobility - walking aid (self-reported), n (%)	
None	8 (8)
Cane	5 (5)
Rollator	69 (65)
Other	10 (9)
Wheelchair (non-ambulatory)	15 (14)
De Morton Mobility Index	53 ± 12 (20–85)
Falls Efficacy Scale	42 (28–56)
Performance Oriented Mobility Assessment	20 (16–24)
Functional Ambulation Categories	4 (3–4)
Gait speed (m/s)^1^	0.57 ± 0.20 (0.22-1.43)
6 Minute Walk Test (m)	153 ± 86 (0–454)

Values are presented as mean ± standard deviation (range), median (interquartile range).

^1^n = 99.

Scores on interval based measures were not normally distributed (DEMMI: W = 0.968; *P* = 0.011; gait speed: W = 0.942, *P* < 0.001; 6MWT: W = 0.947, *P* = 0.001). Table 3 shows Spearman’s correlation coefficients between DEMMI scores and scores of other outcome measures. The point estimate of the correlation between DEMMI scores and gait speed was slightly below that hypothesised (rho = 0.67, 95% CI: 0.54 to 0.76).

**Table 3:** **Spearman’s correlation coefficients between DEMMI and other outcome parameters**

**Construct**	**Measure**	**rho**	**95% CI**
Mobility and fall risk	Performance Oriented Mobility Assessment	0.89*	0.84 to 0.92
Ambulation	Functional Ambulation Categories	0.70*	0.59 to 0.78
Gait speed^1^	10 Meter Walk Test	0.67*	0.54 to 0.76
Walking capacity	6 Minute Walk Test	0.73*	0.62 to 0.80
Fear of falling	Falls Efficacy Scale International	−0.68*	−0.77 to −0.56
Comorbidity	Charlson Comorbidity Index (age adjusted)	−0.03	−0.22 to 0.16

CI = confidence interval, ^1^n = 99, *indicates p < 0.001.

As our sample included most solely cognitively intact older individuals (MMSE IQR: 27 – 30 points), any correlational analysis on divergent validity between DEMMI and MMSE scores is inadequate due to the narrow MMSE range and was not performed. DEMMI scores did not correlate significantly with CCI scores.

DEMMI scores differed significantly between the three groups categorized by the level of ambulation (H[3] = 40.0, *P* < 0.001). Participants who were able to ambulate in the hospital independently (n = 85; 57 ± 9) had higher DEMMI mean scores (Mann–Whitney *U* = 58; *P* < 0.001) than the participants who needed physical support or supervision from an assisting person (n = 8; 41 ± 7; difference between the means: 16 points). However, those participants did not perform significantly better (Mann–Whitney *U* = 40; *P* < 0.27) than participants who were non-ambulatory (n = 14; 35 ± 10). As expected there was a significant mean difference of 14 points (Mann–Whitney U = 129; P = 0.001) in DEMMI scores of participants who needed a walking aid or a wheelchair (n = 99; 52 ± 12) and those who walked without a walking aid (n = 8; 66 ± 10).

### Internal consistency

Internal consistency was α = 0.83 (evaluated based on the validity analysis sample).

### Rasch analysis

The pooled sample for the Rasch analysis consisted of 140 sub-acute geriatric patients (66% female) with a mean age of 79.7 ± 6.3 years and a mean DEMMI score of 52 ± 13 points. All participants were able to sit unsupported for 10 seconds (item 4), and this extreme item was therefore excluded from the analysis.

Overall fit to the model of the remaining 14 items was achieved with a non-significant (Bonferroni adjusted *P* = 0.05/14 = 0.004) chi-square value (44.45, df = 28, *P* = 0.025). The data were confirmed as meeting the assumption of local independence. The residual correlations for items 9 and 10, and 7 and 8 had correlations greater than 0.2 (0.29 and 0.34 respectively), but subtest analysis showed the person separation index (0.84) and Cronbach’s alpha (0.85) were unchanged from the full item set. Unidimensionality was confirmed with only 2.87% of the sample returning significantly different person location values on the 4-item subsets formed from the residual principle component loadings.

There were no disordered thresholds or misfitting items, and no DIF by age, gender or age adjusted CCI.

**Figure 3:**
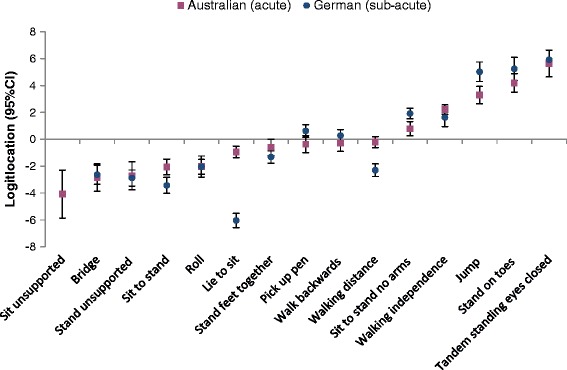
Item logit location. Item logit location (with 95% confidence intervals) and item hierarchy of difficulty of the German and Australian [20] DEMMI data. Item “sit unsupported for 10 seconds” excluded from the German analysis.

Figure 3 shows the item hierarchy of the German DEMMI (aged rehabilitation sample) compared to the original Australian English DEMMI (developed in an Australian aged acute hospitalized population) version. A high positive logit location (e.g. standing on toes) indicates harder item difficulty compared to a negative logit location (e.g. bridging). There was some deviation from the original hierarchy. In the rehabilitation sample, item 3 (lie to sit), item 5 (sit to stand) and item 11 (walking distance) were easier and items 6 (sit to stand no arms) and item 15 (jump) were more difficult than for the acute sample, with non-overlapping 95% confidence bands.

## Discussion

This is the first study that has examined the reliability and validity of the German translation of the DEMMI used by physiotherapists in a population of patients admitted to a sub-acute geriatric inpatient rehabilitation hospital. To review the success of cross-cultural validation, it is crucial to compare the results found in this study to the results of other studies on the DEMMI’s psychometrics.

### Comparison with other studies

Two studies examined the DEMMI’s psychometrics in Australian older people receiving inpatient rehabilitation [25,53]. Mean age (81.8 and 83.4 years, respectively) and proportion of female participants (57.1% and 76%, respectively) was comparable to our sample. However, MMSE (mean 24.0 points) and CCI (mean 1.3) scores were lower in the one study these variables were assessed [53]. Most participants in our study were assessed in the middle or at the end of their rehabilitation. When this data is compared to discharge values reported in the other studies, DEMMI scores (mean: 41 and 49 points, respectively [25,53]) of participants included in this study (mean: 53 points) are even higher, but walking abilities (6MWT: 155 m, gait speed: 33 m/min (approximately 0.54 m/sec) [53]) are comparable. We thus acknowledge the limited comparability between these samples.

Inter-rater reliability between two trained physiotherapists was excellent (ICC = 0.94), with good agreement in most of the 15 DEMMI items. This result shows high accordance with other reliability studies that found comparable reliability indexes in acute medical patients (r = 0.92) [24] and in a sub-acute geriatric rehabilitation setting (r = 0.87) [25].

The absolute reliability (MDC_90_ of 9 points) indicates that there must be a change score of at least 9 points for an assessor to be 90% confident that a true change has occurred. This value is similar to the values reported in other trials, where the MDC_90_ was between 8 and 10 points in acute and sub-acute geriatric inpatients [24,25].

Convergent construct validity was indicated by confirmation of the hypotheses of strong correlations between DEMMI scores, as a measure of mobility, and scores of other mobility related outcome measures. The point correlation between DEMMI scores and gait speed (rho = 0.67) was only slightly lower than expected. These findings are congruent with other trials, where correlations with the 6MWT and gait speed were quite similar in the sub-acute setting [25] or in older patients with hip and knee ostreoarthritis [27].

Known-groups validity was evident with respect to dependence in ambulation and walking aid use. The fit of the data for 14 items in the German DEMMI version to a Rasch model confirmed that it is a unidimensional scale. There were, however, some differences in the average location of several items on the logit scale. In the current study the easiest of the 14 analysed items were 3 (lie to sit) and 5 (sit to stand) compared to 1 (bridge) and 7 (stand unsupported) in the comparison study. The most difficult items were 9 (stand on toes) and 10 (tandem stand eyes closed), a result similar to both the Australian [20] and Dutch [27] samples.

### Strengths and limitations of study

The sample size of the reliability study was as large as calculated *a priori* and it is comparable to previous examinations on the DEMMI’s reliability [20,25,27]. The 95% CI was narrower than expected (0.88 to 0.97) and the lower limit of the 95% CI of the ICC (0.88) is higher than the recommended minimum standard of reliability (ICC ≥ 0.70) [50]. However, inter-rater reliability was only assessed between 2 trained raters with a quite similar level of work experience. One can assume that the inter-rater reliability in a larger sample of raters in clinical practice would be comparable if the same learning procedure is followed. However, reliability studies between more diverse raters are desirable and would provide reliability estimations with higher external validity.

The calculation of the MDC followed the approach described by Stratford et al. [48]. Therefore, data of stable patients is needed to detect measurement error over time (longitudinal approach) [47,48]. The MDC calculation performed in this study includes the ICC between 2 assessors found in this study. As both assessments were performed during a short period of time (30 minutes), the ICC includes the inter-rater variance (between both raters) and the participant’s intra-individual variance (test-retest between both time points). Thus, the MDC of 9 points of the German DEMMI version in the sub-acute geriatric setting might be biased by the short period between both assessments and the inter-rater variance included in the ICC value. Further research should use reliability data of stable patients assessed over a longer period relevant for the inpatient rehabilitation setting (e.g. 2 to 3 weeks) by one single rater to further prove the MDC found in this study.

For the convergent validity analysis, we used only one assessment (POMA) that contained multiple components of mobility. The others (FAC, 6MWT, gait speed) are actually measures of ambulation and gait, and do not rate bed mobility, transfer abilities and higher levels of functional ambulation. The FES-I is only an indirect perception of mobility as it rates fear of falling.

Divergent validity based on DEMMI and MMSE correlations could not be analysed as intended in the study protocol because the included participants did not cover the potential width in MMSE scores. However, the DEMMIs discriminant validity was indicated by a non-significant and low correlation with the CCI and was further proven in another study reporting a weak correlation (0.24) with the MMSE [20].

The DEMMI was developed in a consecutive acute medical sample [20]. As we used a cross-sectional design, most sub-acute participants were already an inpatient for some time (13 ± 6 days) and thus presented with a higher mobility level than on admission. That is why we abstained from the calculation of floor and ceiling effects. In the convenient validation sample, assessments were performed by only 3 physiotherapists differing in their level of working experience. This may not entirely represent the real life clinic and its variety of raters and patients, and thus, evidence for the psychometric characteristics of the DEMMI in daily clinical practice is limited. However, in the first study on cross-cultural adaption [28], the German DEMMI version was performed by a complete clinical section of physiotherapists in 133 consecutive geriatric inpatients, and by doing so no floor- or ceiling effects were observed. Thus, there is evidence for the DEMMI to overcome crucial issues with floor and ceiling effects [20,24,27,28] that hamper clinical interpretability of most common mobility measurement instruments reported in other studies [16-18].

Rasch analysis could only be performed on a 14-item DEMMI as item 4 (sit unsupported) could be performed by all 140 participants. This can be explained by the higher functional abilities of the present convenient sample due to the later recruitment in this study, in contrast to the Australian development sample [20]. For that reason we kept item 4 (sit unsupported) in the German DEMMI version. Furthermore, this item gives clinically relevant information on the functioning of an elderly patient and it was also the easiest item in the Australian English and the Dutch DEMMI versions [20,27]. However, a further study should include a consecutive sample of sub-acute geriatric inpatients who perform the DEMMI immediately after hospital admission.

When it comes to clinical implementation of the DEMMI in a team of health care professions, a short learning phase seems essential to gain such reliable scores between raters. We recommend the approach described in this study, to use the German instruction handbook and to pay attention especially to these items that showed lower agreement in the present study (see Table 2, eg. “lying to sitting”). Cognition can have a significant impact on mobility [56,57]. In the current study, patients with low MMSE scores were excluded. However, the high proportion of cognitively impaired patients in geriatric inpatient settings is clinically very important [58]. These patients were not specially excluded in the general geriatric acute medical DEMMI development sample (mean MMSE: 21.7 ± 7.6, range 0–30) [20]. Through the successful cross-cultural validation process, the German DEMMI seems to be a valid measure of mobility for patients presenting with various cognitive abilities. However, the DEMMI’s psychometric properties solely in the considerable population of older individuals with cognitive impairment need to be further examined.

The DEMMI is considered to measure a patient’s mobility in various settings and with various disease conditions where mobility functions of older people are an important indicator of independence in the ADLs, quality of life and health status. Thus, more research is needed on the German DEMMI version in acute [20] and community-dwelling [59] older people, as well as in nursing home residents.

Mobility can also be affected crucially in several geriatric diseases and syndromes, such as osteoarthritis [27], Parkinson’s disease [26], hip-fracture [53], stroke, frailty, dementia or chronic obstructive pulmonary disease. We recommend further psychometric examination in these conditions, including analysis of responsiveness, interpretability and prognostic validity of the German DEMMI translation.

## Conclusions

The German translation of the DEMMI is a valid measure of the mobility of older individuals in sub-acute geriatric inpatient care and measurement error between two physiotherapists is acceptably small. Results in this study were consistent with the ones found for the English original version in an Australian population, indicating a successful cross-cultural adaption. Considering its feasibility and simplicity, the DEMMI can be implemented into clinical practice to measure the mobility status of geriatric inpatients.

## Abbreviations

6MWT: Six-minute walk test
ADL: Activities of daily living
CCI: Charlson comorbidity index
CI: Confidence interval
DEMMI: De Morton mobility index
DIF: Different item functioning
FAC: Functional ambulation categories
FES-I: Falls efficacy scale international
ICC: Intraclass correlation coefficient
ICF: International classification of functioning
MCID: Minimal clinical important difference
MDC90: Minimal detectable change with 90% confidence
MMSE: Mini mental state examination
POMA: Performance oriented mobility assessment
SD: Standard deviation
SEM: Standard error of measurement
